# Dissecting the Genetic Basis of the Technological, Functional, and Safety Characteristics of *Lacticaseibacillus paracasei* SRX10

**DOI:** 10.3390/microorganisms12010093

**Published:** 2024-01-02

**Authors:** Christina S. Kamarinou, Despoina E. Kiousi, Panagiotis Repanas, Anthoula A. Argyri, Nikos G. Chorianopoulos, Alex Galanis

**Affiliations:** 1 Department of Molecular Biology and Genetics, Faculty of Health Sciences, Democritus University of Thrace, 68100 Alexandroupolis, Greece; christinakamarinou7@gmail.com (C.S.K.); dkiousi@mbg.duth.gr (D.E.K.); panrepa@hotmail.com (P.R.); 2 Institute of Technology of Agricultural Products, Hellenic Agricultural Organization-DIMITRA, 14123 Lycovrissi, Greece; anthi.argyri@elgo.gr; 3 Laboratory of Microbiology and Biotechnology of Foods, School of Food and Nutritional Sciences, Department of Food Science and Human Nutrition, Agricultural University of Athens, 11855 Athens, Greece; nchorian@aua.gr

**Keywords:** nonstarter lactic acid bacteria, *Lacticaseibacillus paracasei*, whole-genome sequencing, metabolic capacity, multiplex PCR

## Abstract

Nonstarter lactic acid bacteria (NSLAB) are major contributors to the unique characteristics (e.g., aroma, flavor, texture) of dairy and nondairy fermented products. *Lc. paracasei* SRX10 is an NSLAB strain originally isolated from a traditional Greek cheese and previously shown to exhibit favorable biotechnological characteristics. More specifically, the strain showed tolerance to simulated gastrointestinal conditions, exopolysaccharide (EPS) biosynthetic capacity, and lack of hemolytic activity and was used in the production of yoghurt and feta cheese with distinct organoleptic characteristics. The aim of the present study was to investigate these traits at the genome level through whole-genome sequencing (WGS), annotation, and comparative genomics. Functional annotation of the genome revealed that *Lc. paracasei* SRX10 can utilize different carbon sources, leading to the generation of flavor compounds, including lactic acid, acetate, ethanol, and acetoin. Similarly, full clusters for fatty acid biosynthesis, protein and peptide degradation, as well as genes related to survival under extreme temperatures, osmotic shock, and oxidative stress were annotated. Importantly, no transferable antibiotic resistance genes or virulence factors were identified. Finally, strain-specific primers based on genome-wide polymorphisms were designed for the efficient and rapid identification of *Lc. paracasei* SRX10 via multiplex PCR in fermented products.

## 1. Introduction

Lactic acid bacteria (LAB) play a vital role in the development of cheese flavor, serving as a starter or nonstarter LAB (NSLAB) culture during curd acidification and ripening [1,2]. NSLAB occur naturally as environmental contaminants in raw milk at initially low concentrations; eventually, they become the dominant microbial population in ripened cheeses, as they show resistance to chemical and physical stresses [3]. Facultative heterofermentative lactobacilli represent a predominant group among NSLAB [4]. Within this group, the genus *Lacticaseibacillus*, consisting of the species *Lacticaseibacillus paracasei*, *Lc. casei*, and *Lc. rhamnosus*, is particularly prevalent in long-ripening cheeses, and its contribution to flavor development has been recognized [2,5]. NSLAB possessing unique functional characteristics and technological properties can be used as adjunct cultures in fermented foodstuffs [6,7]. More specifically, NSLAB can contribute to the development of unique sensory characteristics by positively influencing aroma, flavor, and texture. Moreover, NSLAB can be exploited to produce foods rich in vitamins, cofactors, and amino acids [8,9].

Increased accessibility to next-generation sequencing (NGS) platforms has enabled in silico investigation of the unique genome-wide technological and functional properties of NSLAB [10]. In this context, food safety agencies, including the European Food Safety Authority (EFSA), require whole-genome sequencing (WGS) of strains intended for use in the food chain to provide convincing evidence for their taxonomic classification, as well as information on their functional, technological, and safety properties [11]. To this end, in silico pipelines and bioinformatic algorithms have been developed to predict the metabolic and biosynthetic capacity of LAB [12]. Furthermore, safety assessments can be performed based on the presence of transferable elements, including antibiotic resistance genes, virulence factors, and toxins [13,14]. Importantly, genome-wide polymorphisms in strains of interest can be used for their efficient detection in complex multimicrobial matrices. Indeed, previous studies have exploited unique species- or strain-specific genetic regions to develop PCR-based detection protocols [15,16]. In particular, regulatory authorities require accurate labeling of functional foods, which includes proper identification and quantification of the strains present [17].

*Lc. paracasei* SRX10 is a LAB strain originally isolated from fresh semihard goat cheese [7]. Previous in vitro tests have shown that *Lc. paracasei* SRX10 exhibits favorable technological and functional properties, including tolerance to acidic pH and bile salts, susceptibility to common antibiotics, and the ability to produce β-galactosidase and exopolysaccharides [7]. Furthermore, the strain was successfully incorporated as an adjunct culture in the production of yogurt and feta cheese, with desirable and distinctive organoleptic characteristics [7,18]. The aim of this study was to explore the genetic basis of these properties using comprehensive bioinformatic analysis. More specifically, the genome of *Lc. paracasei* SRX10 was sequenced, annotated, and searched for the presence of genes involved in tolerance to fermented food industry conditions (e.g., oxidative and osmotic stress). In addition, metabolic pathways were annotated to examine the capacity of the strain to produce compounds and secondary metabolites that contribute to the aroma and flavor of fermented foods. Furthermore, the safety profile of the strain was assessed via evaluating genome stability, the ability to produce toxins and virulence factors, and the presence of transferable antibiotic resistance genes. Finally, comparative genomic analysis was performed to identify unique genomic regions that served as templates for the design of strain-specific primers for the efficient detection of *Lc. paracasei* SRX10 in fermented foods using single-step multiple PCR.

## 2. Materials and Methods

### 2.1. Bacterial Strains, Culture Conditions, and DNA Isolation

*Lc. paracasei* SRX10 was previously isolated from a traditional Greek cheese [7]. *Lc. paracasei* SP3 and *Lc. paracasei* SP5 were isolated from kefir grains [19,20], and *Lp. plantarum* L125 was isolated from fermented sausages [21]. Strains *Lc. rhamnosus* GG and *Lc. casei* ATCC 393 were purchased from ATCC (LGC Standards, Middlesex, UK) and *Lc. paracasei* DSM 20,006 was obtained from DSMZ (Braunschweig, Germany). All strains were maintained in de Man, Rogosa, and Sharpe (MRS) broth (Condalab, Madrid, Spain) at 37 °C in anaerobic conditions. For DNA extraction, bacterial cells were harvested via centrifugation (8000× *g* for 4 min). The cell pellet was lysed, and DNA was extracted using the NucleoSpin^®^ Tissue kit, following manufacturer’s instructions (Macherey-Nagel, Düren, Germany). DNA purity and quantity were determined spectrophotometrically (NanoDrop^®^ ND-1000 UV–Vis Spectrophotometer, Thermo Fisher Scientific, Waltham, MA, USA).

### 2.2. Whole-Genome Sequencing, Assembly, and Annotation

The isolated DNA of *Lc. paracasei* SRX10 was sequenced using the Illumina NovaSeq6000 (2 × 151 paired ends) platform. A total of 10,117,664 paired-end reads were generated. The whole-genome sequence of the strain was constructed de novo and annotated, as previously described [14]. Contamination levels of the genomic sequence were assessed with CheckM v1.0.7 [22]. Finally, Artemis (version 18.1.0), a Java-based software [23], was used to visualize the genome assembly.

### 2.3. Comparative Genomics

Average nucleotide identity (ANI) was used to study the uniqueness and phylogenetic relationships of *Lc. paracasei* SRX10 with dairy-associated *Lc. paracasei* strains. More specifically, genomic FASTA sequences of 109 strains isolated from dairy products were collected from the NCBI Genome database and were used as input in the python module Pyani [24]. Pangenome analysis of this subset was performed with Roary [25], and core genome sequences were used to construct an approximately maximum likelihood phylogenetic tree with FastTree 2.1 [26]. The tree was visualized on the iTOL server [27].

### 2.4. Identification of Genes Involved in Technological and Functional Characteristics

Functional classification of proteins into clusters of orthologous groups (COGs) was executed with the eggNOG-mapper tool (version 2.0), available online at the eggNOG database (version 5.0) [28]. Kyoto Encyclopedia of Genes and Genomes (KEGG) Orthology (KO) assignment of the predicted proteins was performed using BlastKOALA (version 2.2) [29]. Additionally, CAZyme annotation was performed using the dbCAN2 meta server [30].

### 2.5. In Silico Safety Assessment

The safety profile of *Lc. paracasei* SRX10 was investigated in silico, following the guidelines and recommendations of the EFSA FEEDAP Panel [31]. To investigate genome stability, prophage regions were detected using PHAge Search Tool Enhanced Release (PHASTER) [32], insertion sequence elements with ISFinder [33], and clustered regularly interspaced short palindromic repeats (CRISPR) arrays using CRISPRDetect [34] and PILER-CR [35]. The presence of plasmids was predicted using PlasmidFinder [36], and mobile genetic elements were predicted with MobileElementFinder [37]. Genes involved in antimicrobial resistance were annotated using Resistance Gene Identifier (RGI; version 5.2.0) and ResFinder 4.1 [38,39], and the presence of virulence factors and putative pathogenic sequences was determined with VirulenceFinder 2.0 [40,41] and PathogenFinder 1.1 [42], respectively. Finally, the annotated genome sequence was searched for genes involved in hemolysis and biosynthesis of biogenic amines using eggNOG-mapper and Blastp.

### 2.6. Development of a Strain-Specific Multiplex PCR Assay for Detection of Lc. paracasei SRX10 in Monocultures and Yoghurt Samples

Primer design based on unique regions of the WGS of *Lc. paracasei* SRX10 was performed following a previously published methodology [16]. Amplification reactions were performed at a final volume of 20 μL, consisting of 5 units Taq DNA polymerase (Minotech, Heraklion, Greece), 10 mM of each dNTP (Jena Bioscience, Jena, Germany), 1.5 mM MgCl_2_ (Minotech), 1× Taq polymerase buffer (Minotech), and 10 ng of DNA template. Primers were added at a final volume of 1 μL, for a final amount of 25 pmol in each reaction. The universal bacterial primer set LacF/LacR targeting the 16S rDNA gene of lactobacilli [43] served as a positive control. Reactions were carried out in the Veriti thermocycler (Applied Biosystems, Waltham, MA, USA) using the following conditions: 94 °C (1 min), followed by 25 cycles of 94 °C (45 s), 58 °C (30 s), 72 °C (1 min), followed by a final extension step at 72 °C (10 min). The PCR products were separated on 2% (*w*/*v*) agarose gels, visualized under UV illumination, and photographed with a digital camera (Gel Doc EQ System, Bio-Rad, Hercules, CA, USA).

## 3. Results

### 3.1. Genome Features

**Figure 1:**
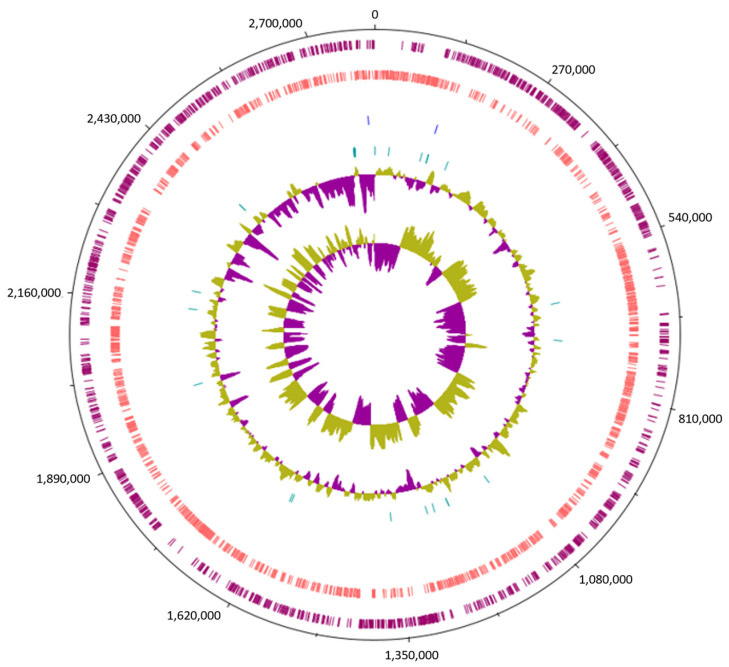
Circular genome map of *Lc. paracasei* SRX10, constructed using Artemis. From outer to inner circle, the genomic features presented are: forward strand CDS (burgundy), reverse strand CDS (pink), rRNA genes (blue), tRNA genes (green), GC content, and GC skew.

The investigation into the genomic features of *Lc. paracasei* SRX10 was conducted through whole-genome sequencing, de novo assembly, and genome annotation (Figure 1). The genome of *Lc. paracasei* SRX10 has a total length of 2,813,407 bp and a GC content of 46.4% (Table 1). The N50 value is 43,810 bp, with the longest contig being 130,527 bp and the mean contig size being 10,572 bp. The completeness of the genome is 99.46%, with a 1.89% level of contamination. The strain harbors 2764 predicted genes, 2711 coding DNA sequences (CDSs), 6 ribosomal RNAs (rRNAs), 44 transfer RNAs (tRNAs), 3 noncoding RNAs (ncRNAs), and 106 pseudogenes (Table 1).

**Figure 2:**
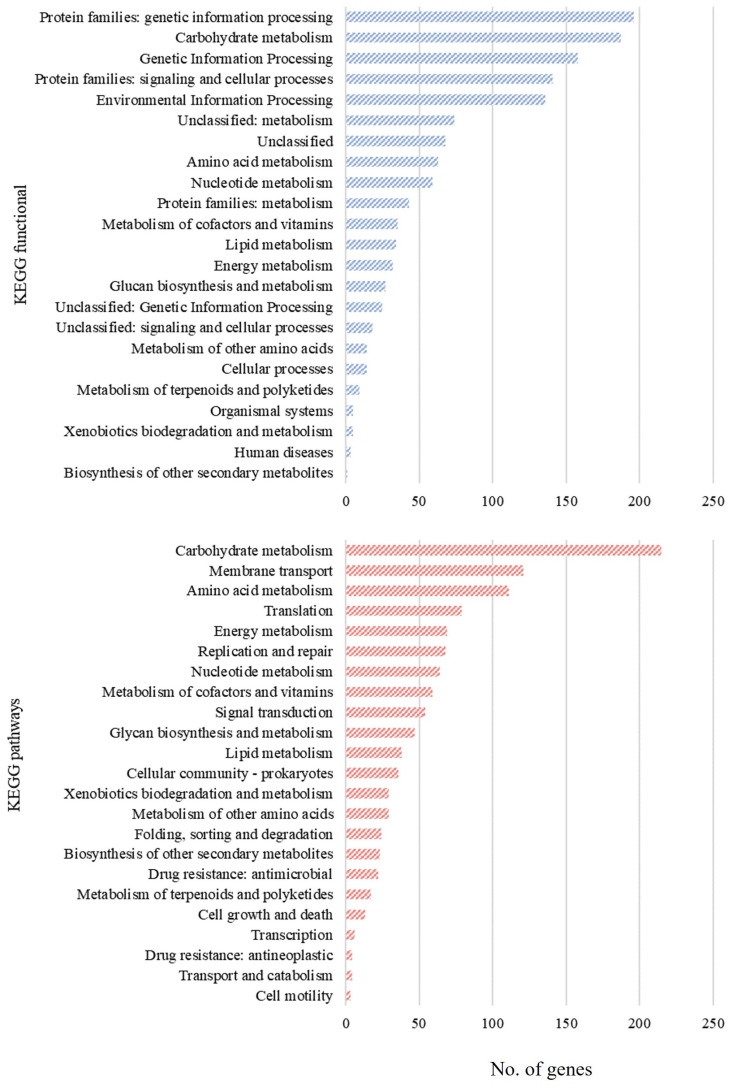
Assignment of annotated genes of *Lc. paracasei* SRX10 to KEGG functional categories and pathways.

The protein sequences of *Lc. paracasei* SRX10 are categorized into 20 COGs. The most represented category is “Function Unknown” (S, 20.37%), followed by “Carbohydrate metabolism and transport” (G, 8.37%), “Transcription” (K, 8.33%), “Amino Acid metabolism and transport” (E, 7.42%), “Translation” (J, 6.62%), and “Replication and repair” (L, 5.98%) (Table S1). Additionally, KEGG assignment to functional categories and pathways led to the classification of predicted proteins into 191 pathways and 23 functional categories (Figure 2). The most represented pathway is “Carbohydrate metabolism” with 215 proteins, followed by “Membrane transport” and “Amino acid metabolism” with 121 and 111 proteins, respectively.

### 3.2. Phylogenomic and Pangenome Analysis

**Figure 3:**
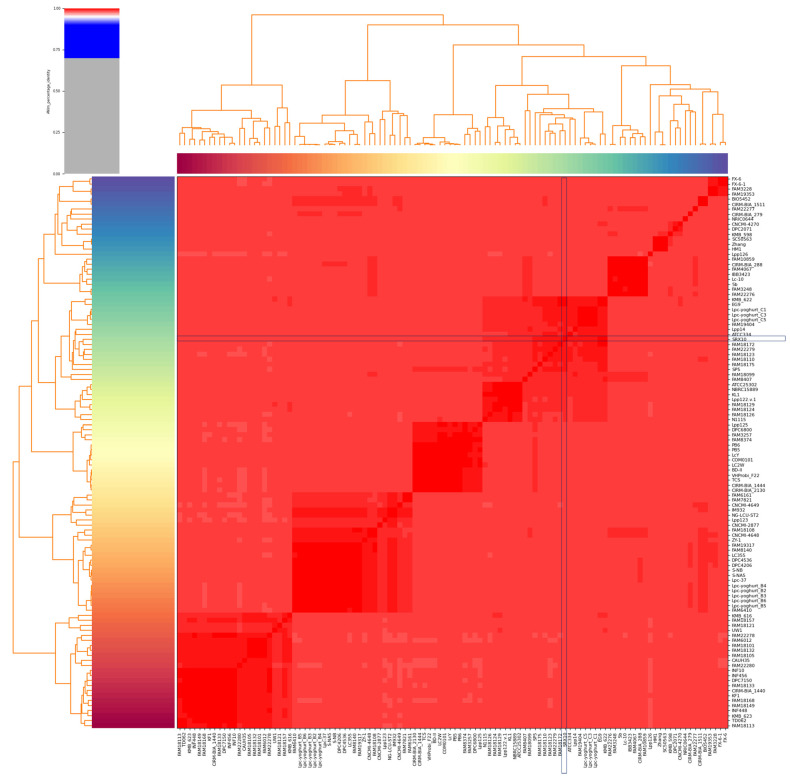
ANI heatmap of *Lc. paracasei* strains derived from dairy products constructed using Pyani (version 0.2.10). The position of *Lc. paracasei* SRX10 is indicated with blue boxes.

**Figure 4:**
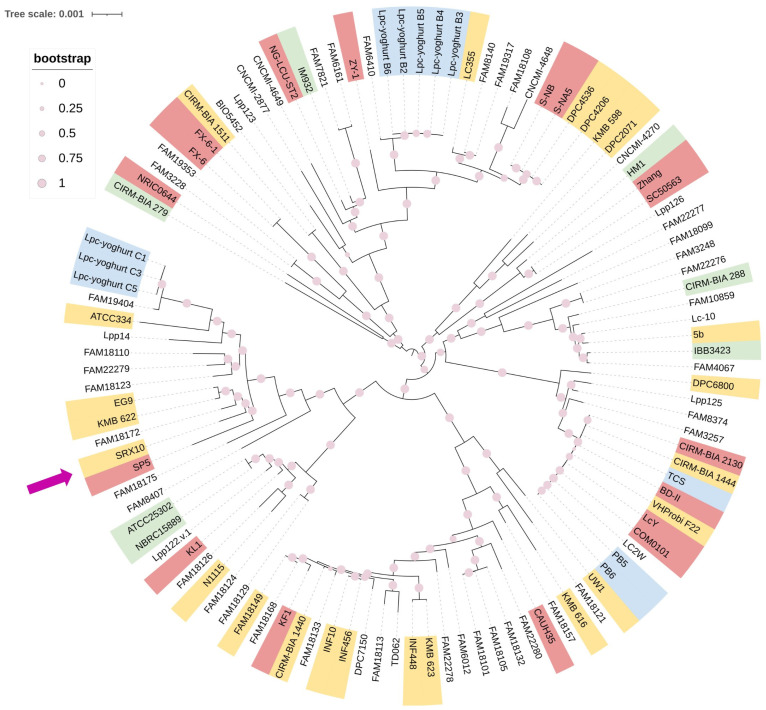
Approximately maximum likelihood phylogenetic tree of *Lc. paracasei* strains isolated from dairy products (pink—fermented milk products, yellow—cheese, blue—yoghurt, green—raw milk products) based on orthologous genes calculated with Roary (version 3.13.0) and built with 1000 bootstrap replications. The purple arrow indicates the position of *Lc. paracasei* SRX10 in the phylogenetic tree.

*Lc. paracasei* SRX10 exhibits a high ANI (>99%) with dairy-associated *Lc. paracasei* strains. Specifically, it presents high genome similarity to *Lc. paracasei* KMB 622 (99.67%), and *Lc. paracasei* EG9 (99.62%), both isolated from cheese products [44,45] (Figure 3, Table 2). Additionally, it clusters with *Lc. paracasei* SP5 [19] and *Lc. paracasei* FAM18172 [45] in a phylogenomic tree constructed using core genes (Figure 4). The pangenome of dairy-associated strains used in the study contains 15,752 orthologous groups. Among these groups, 932 proteins comprise the core genome, 484 comprise the soft-core genome, 2391 comprise the shell genome, and 11,834 proteins comprise the cloud genome (Figure S1).

### 3.3. Detection of Genes Associated with Technological and Functional Characteristics

#### 3.3.1. Stress Tolerance

Genome annotation and comparative bioinformatic analysis were employed to identify genes that may confer resistance to acidic conditions, extreme temperatures, osmotic shock, and oxidative stress. *Lc. paracasei* SRX10 harbors a full gene cluster (*atpABCDEFGH*) for the production of H^+^-transporting ATPase/ATP synthase, which is involved in cytoplasmic pH homeostasis (Table 3). It also codes two cold shock proteins (CspB and CspC), four heat shock proteins (HrcA, Hsp3, Hsp1, DnaK, and GrpE) and five proteins involved in heat shock response: molecular chaperones ClpB and ClpC, nucleotide exchange factor GrpE, chaperonin GroEL, and cochaperonin GroES (Table 3). Additionally, it harbors genes responsible for resistance to osmotic shock, including genes for the production of glycine betaine binding factors (OpuCC, ChoS) and transporters (GbuAB). *Lc. paracasei* SRX10 also codes for peroxidases (Gpo, Tpx, and Ywbn), redox-regulated molecular chaperones (HslO), and NADH oxidases (Ndh and Nox), implicated in the oxidative stress response (Table 3). Finally, three genes (*gabD*, *aldA*, and *gap*) coding for proteins of the aldehyde dehydrogenase family were annotated in the genome of the strain, which can facilitate growth in matrices with elevated ethanol or acetaldehyde content.

#### 3.3.2. Metabolic Pathways and Genes Associated with Flavor and Texture Development

##### Carbohydrate Metabolism

Annotation in the KEGG database showed that *Lc. paracasei* SRX10 possesses full modules for glycolysis (M00001, M00002), gluconeogenesis (M00002), and pyruvate oxidation (M00307), as well as for the production of ribulose 5P through the pentose phosphate pathway (M00006). Complete clusters for galactose degradation (M00632), glycogen/starch production (M00854), and UDP-N-acetyl-D-glucosamine biosynthesis (M00909) were also annotated in the genome of the strain. Additionally, *Lc. paracasei* SRX10 codes for alpha amylase (MalA), which catalyzes starch hydrolysis. A total of 66 genes involved in the metabolism of various carbohydrates were identified utilizing the dbCAN server, which were further categorized into five CAZymes classes. More specifically, genes coding for 30 glycoside hydrolases (GHs), 27 glycosyltransferases (GTs), 2 carbohydrate-binding modules (CBMs), 4 carbohydrate esterases (CEs), and 3 auxiliary activity (AA) genes were annotated (Table S2).

*Lc. paracasei* SRX10 codes for multiple carbohydrate uptake systems, including the LacEF phosphotransferases (PTSs) for lactose, galactose, cellobiose, and β-galacto-oligosaccharides; SrlB, a PTS system for the transport of glucitol, sorbitol, and fructose; and sugar-specific permeases. The LacFE/LacG system encoded by the strain can support the simultaneous utilization of both glucose and galactose. No genes for the production of LacS/LacLM and LacZ, which are responsible for the preferential metabolism of glucose and the excretion of galactose, were identified. The strain can also process galactose through the products of the *galETKM* operon via the Leroir pathway (Table 4). Glucose-1P is then transformed into glucose-6P, a glycolysis intermediate, by phosphoglucomutases that belong to the phosphoexose mutase family. Furthermore, lactose is processed through the lacTEGF operon for the production of tagatose-1,6P, which serves as a substrate for tagatose-1,6P aldolase LacD that leads to the generation of glyceraldehyde 3-phospate, an intermediate substrate for glycolysis. Subsequently, pyruvate is produced through the Embden–Meyerhof pathway (M00001). *Lc. paracasei* SRX10 also codes for the FMN-dependent L-lactate dehydrogenase LctO and for D-lactate dehydrogenase dld, which catalyze the conversion of pyruvate to lactic acid. Lactic acid is the primary product produced via the metabolism of lactose by LAB during fermentation, which is responsible for matrix acidification, as well as for the distinctive flavor of fermented foods. Importantly, lactic acid limits the growth of spoilage microorganisms, enhancing shelf life and the safety of fermented foodstuffs. Moreover, a portion of the intermediate pyruvate can undergo alternative metabolic pathways to support pH homeostasis in acidic conditions, leading to the production of flavor compounds like diacetyl, acetoin, acetaldehyde, or acetic acid. To this end, *Lc. paracasei* SRX10 codes for a pyruvate dehydrogenase that catalyzes the production acetyl-CoA, subsequently leading to the formation of acetate or ethanol. On the other hand, the strain does not possess an α-acetolactate synthase that catalyzes the transformation of pyruvate to α-acetolactate, a precursor of diacetyl and acetoin. Finally, the strain codes for BudA, an α-acetolactate decarboxylase, which is responsible for the production of acetoin from α-acetolactate.

##### Lipid Metabolism

Fatty acid biosynthesis and degradation pathways are vital for the formation of aroma compounds, thus significantly contributing to cheese flavor [46]. Concerning the KEGG pathway assignment of *Lc. paracasei* SRX10, three annotated proteins (K00001, K00626 and K04072) cluster in “Fatty acid degradation” (KO 00071), and 10 annotated proteins (K00059, K00645, K00648, K01961, K01962, K01963, K02160, K02371, K02372, and K09458) cluster in “Fatty acid biosynthesis” (KO 00061). More specifically, *Lc. paracasei* SRX10 carries genes *fabH*, *fabD*, *fabF*, *fabG*, *fabK*, and *fabZ* and the cluster *accABCD* for saturated and unsaturated fatty acid production. Furthermore, it codes for two phospholipases that catalyze the formation of fatty acids from phospholipids. In this context, *Lc. paracasei* SRX10 codes for a GDSL-like lipase/acylhydrolase and for triacyglycerol lipase (LipA), which catalyze the hydrolysis of a diverse set of lipidic substrates, including phospholipids, thioesters, and triglycerides. Fatty acids can be further processed to generate aromatic, volatile compounds including fatty acid derivatives (esters and thioesters), γ- or δ-lactones, and aldehydes [47]; however, the strain lacks full operons for their production.

##### Protein Metabolism

Proteolysis in LAB is crucial for the acquisition of amino acids that cannot be synthesized by the cell. To this end, *Lc. paracasei* SRX10 contains full clusters only for the production of 5 out of the 20 amino acids (threonine, cysteine, lysine, proline, and histidine). Two dipeptide transporter systems were identified in the genome of the strain: oligopeptide transport ATP-binding protein (Opp) and the amino acid transporter DtpT. Numerous proteases and peptidases were annotated in the genome of strain. In more detail, *Lc. paracasei* SRX10 codes for endopeptidases (PepE), proline peptidases (PepI, PepQ, PepR, and PepX), aminopeptidases (PepN and PepC), oligopeptidases (PepF and PepF2) dipeptidases (PepD2, PepD3, PepDA, PepD, and PepV), and the tripeptidase PepT (Table 4). Branched-chain amino acids (valine, leucine, and isoleucine), the aromatic amino acids (tyrosine, tryptophan, and phenylalanine), and the sulfur-containing amino acids (methionine and cysteine) are the main amino acid precursors of flavor compounds [48]. Specifically, *Lc. paracasei* SRX10 codes for the aminotransferases and dehydrogenases (e.g., Shikimate dehydrogenase) that catalyze the conversion of branched-chained and aromatic amino acids into α-keto acids, which can subsequently lead to the formation of aromatic aldehydes, esters, and thioesters.

##### EPS Production

The genome of *Lc. paracasei* SRX10 was searched for genes *epsA*, *epsB*, *epsC*, *epsD*, *wzy*, and *wzx* (Table 4). To this aim, the sequence of homologous proteins identified in *Lc. paracasei* genomes was derived from UniProt and used for local Blastp. *Lc. paracasei* SRX10 codes for CpsD/CapB-family tyrosine-protein kinase, which presents 80% sequence identity and 90% positive substitutions with EpsB; and for Wzz/FepE/Etk N-terminal domain-containing protein, which possesses 57% sequence identity and 80% positive substitutions with EpsC. Furthermore, it encodes for two glycotransferases: the first (SRX10_002122) presents 60% sequence identity and 79% positive substitutions with EpsD, and the second (SRX10_002125) presents 99% sequence identity and positive substitutions with EpsE. No proteins with significant similarity to EpsA, Wzy, or Wzx were identified in the genome of the strain.

### 3.4. Investigation of Genomic Features Related to the Safety Profile of Lc. paracasei SRX10

#### 3.4.1. Genome Stability

Three prophage regions were predicted in the genome of *Lc. paracasei* SRX10 using PHASTER. Among those, two were found to be intact, and one was categorized as questionable, while none of the identified regions were considered incomplete (Table S3). No plasmids or mobile genetic elements were detected, while a total of 98 insertion elements were identified in the genome of the strain (Table S4). These insertion elements originate from other LAB prevalent in the fermented food industry, including *Lc. casei*, *Lc. rhamnosus*, *Lp. plantarum*, and *Pediococcus pentosaceus*. Finally, *Lc. paracasei* SRX10 lacks functional CRISPR arrays and does not code for Cas proteins (Table 5).

#### 3.4.2. Virulence and Antibiotic Resistance

Bioinformatic analysis using the VirulenceFinder tool showed that *Lc. paracasei* SRX10 does not code for virulence factors (Table 5). However, a gene for a hemolysin III family protein was annotated using eggNog, PROKKA, and PGAP. Further analysis with InterPro showed that the protein carries conserved transmembrane helices characteristic of the hemolysin family (Figure S2). No acquired antibiotic resistance genes were identified using RGI or ResFinder. Nonetheless, chromosomally encoded genes responsible for vancomycin resistance (*vanR*, *vanZ*) were identified in the genome of the strain. Furthermore, a gene coding for the small multidrug resistance protein, SugE, was annotated using eggNOG-mapper.

#### 3.4.3. Biogenic Amine Production

The genome of *Lc. paracasei* SRX10 was examined for genes responsible for the production of biogenic amines (spermidine, cadaverine, putrescine, ornithine, histamine, tyramine, tryptamine, and agmatine) using annotation algorithms. It was confirmed that *Lc. paracasei* SRX10 does not harbor genes encoding the enzymes spermidine synthase (EC 2.5.1.16), ornithine decarboxylase (EC 4.1.1.17), lysine decarboxylase (EC 4.1.1.18), arginine decarboxylase (EC 4.1.1.19), arginase (EC 3.5.3.1), agmatinase decarboxylase (EC 3.5.3.11), histidine decarboxylase (EC 4.1.1.22), tyrosine decarboxylase (EC 4.1.1.25), or tryptophane decarboxylase (EC 4.1.1.28).

### 3.5. Development of a Strain-Specific PCR Assay for Lc. paracasei SRX10 Using Whole-Genome-Based Primers

**Figure 5:**
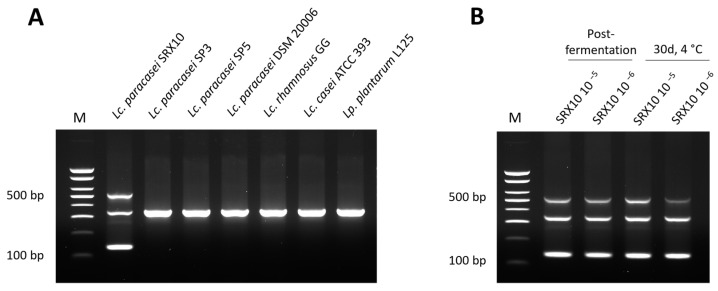
Electrophoretic profile generated with the two specific primer sets and the universal bacterial primer set LacF/LacR in triplex PCR with gDNA derived from *Lc. paracasei* SRX10 and other LAB in monocultures (**A**) or in yogurt samples (**B**). M: 100 bp DNA ladder.

Whole-genome-based primers were designed for *Lc. paracasei* SRX10, following a recently published protocol [16]. Genome-wide comparative genomic analysis between the strain of interest and members of the same species was pursued to identify unique sequences. Upon filtering, five contigs were chosen as templates for primer design. Fifty primer pairs were generated, amongst which two were selected for in vitro validation based on their specificity and capacity to generate a unique electrophoretic pattern for the strain of interest in multiplex PCR (Table 6). As shown in Figure 5A, the pattern consists of a 534 bp band derived from the primer set 1.1F/1.1R, a band at 137 bp generated by the primer set 3.1F/3.1R, and a band at 340 bp generated by the universal bacterial primer set LacF/LacR [43]. This pattern was also replicated for DNA isolated from yogurt samples containing *Lc. paracasei* SRX10 at two different concentrations (5 and 6 log CFU/g) and at two different timepoints: immediately after fermentation and after a 30-day storage at 4 °C (Figure 5B).

## 4. Discussion

In this study, whole-genome sequencing, annotation and comprehensive bioinformatic analyses were performed to provide deeper understanding of the functional, technological, and safety properties of *Lc. paracasei* SRX10, a multifunctional strain that has previously been employed as an adjunct culture in yoghurt and feta cheese production [7,18]. The genome of the strain consists of a chromosome with a length of 2.8 Mb and a GC content of 46.4%, coding for 2711 putative proteins. *Lc. paracasei* strains possess a median genome length of 3.01 Mb and a median GC content of 46.3% (available online: https://www.ncbi.nlm.nih.gov/genome/?term=txid1597[orgn]&shouldredirect=false, accessed on October 2023). Members of the emended *Lactobacillus* genus carry genomes that vary in size, ranging from 1.27 to 4.91 Mb [49], with a variable number of coding sequences [50]. Free-living and nomadic strains possess large genomes, ranging between 3 and 4 Mb, while strictly host-associated strains tend to possess genomes at the lower end of the spectrum [49]. The genome metrics of *Lc. paracasei* SRX10 support its classification into the nomadic *Lc. paracasei* species. Further phylogenomic analysis ensued to confirm the taxonomy of the novel strain. Specifically, *Lc. paracasei* SRX10 exhibits high ANI with other members of the *Lc. paracasei* species (>96%, species threshold). Indeed, *Lc. paracasei* SRX10 exhibits a high degree of similarity with *Lc. paracasei* KMB_622 (99.67%) and *Lc. paracasei* EG9 (99.62%), both isolated from cheese [51]. These findings align with the initial classification of the strain in the *Lc. paracasei* species, which was performed using 16S rRNA sequencing and species-specific multiplex PCR assays [7].

Strain viability in functional fermented products should be ensured through the various stages of production. The pasteurization of dairy products, low temperature during transport and storage, and exposure to oxygen and alcohol can significantly compromise the viability of starter and adjunct cultures, while osmotic stress during production is a widely used method to stop the growth of spoilage and pathogenic microorganisms [52]. However, LAB tend to exhibit greater tolerance to these stresses, which makes them valuable contributors to the fermentation process and the preservation of various foods [53]. Here, we annotated several genes involved in survival under these harsh conditions (Table 3), in agreement with previous data on the viability of the strain during yoghurt and feta cheese production [7,18]. Numerous studies have also identified these loci within the genome of the *Lc. paracasei* strains that are utilized in food fermentation, including *Lc. paracasei* NFBC 338, *Lc. paracasei* UNQLpc 10, and *Lc. paracasei* SP5 [14,54,55]. Concomitantly, they can support survival in the gastrointestinal tract. Indeed, it was previously shown that *Lc. paracasei* SRX10 can survive in acidic conditions and in the presence of bile salts [7].

NSLAB can alter the appearance, aroma, flavor, and texture attributes of fermented foodstuffs through the degradation of the food matrix and the production of secondary metabolites [56]. Along this vein, we analyzed the biosynthetic and catabolic ability of *Lc. paracasei* SRX10 in silico. The majority of the annotated proteins participate in carbohydrate metabolism, as evidenced from the KEGG annotation and COG clustering results. Upon further investigation, transport systems for numerous sugars were identified in the genome of the strain. Glucose and lactose utilization systems were also detected. The degradation of lactose, the main sugar found in milk, results in the production of pyruvate and acetyl–CoA via EMP, that constitute the building blocks of flavor compounds (e.g., organic acids and ethanol) [57]. Organic acids, including lactic acid, formic, and acetic acid, are the main contributors to the acidification of the food matrix and to the distinct aroma and flavor of fermented dairy products [58]. Previously, we showed that lactic acid was produced in high concentrations in feta cheese fermented by *Lc. paracasei* SRX10 (16.3–22.0 mg/g), while citric acid and acetic acid were detected in lower quantities [18]. Lipid and protein degradation in the food matrix during production and ripening is known to determine the nature of the final product [59,60]. Indeed, the lipid and glyceride profile of end products varies among different types of fermented dairy foods [61]. *Lc. paracasei* SRX10 possesses full modules for fatty acid biosynthesis and degradation and codes for multiple proteins for peptide transport, degradation, and amino acid metabolism. Further analysis with chromatographic methods is required to determine the capacity of the strain to produce aromatic lipids and amino acids that can affect the sensory characteristics of different food matrices. The biosynthesis of EPS in the dairy matrix contributes significantly to the appearance, stability, and rheological properties of foods [62]. Indeed, starter cultures containing EPS-producing bacteria, including members of the *Lc. paracasei* species, have been incorporated in the production of yoghurt, cheese, kefir, as well as in the development of nondairy products [63]. Here, an EPS-encoding cluster containing genes *epsD*, *epsB*, and *epsG* was annotated, in agreement with previous in vitro findings [7]. Future studies will focus on the characterization of the secondary metabolites secreted by *Lc. paracasei* SRX10 in situ using metabolomic and proteomic platforms, focusing on its ability to synthesize vitamins, coenzymes, and antimicrobial peptides, to establish its potential health-related properties.

In this study, the safety profile of *Lc. paracasei* was investigated in silico, complimenting previous in vitro findings [7]. Initially, the genome stability of the strain was examined by investigating the presence of plasmids, insertion, and mobile elements using annotation algorithms. The genomic and phenotype stability of the strains is a prerequisite for their safety and functionality for use in the fermented food industry [64]. To this end, genome analysis revealed that *Lc. paracasei* SRX10 lacks plasmids, mobile elements, and functional CRISPR arrays; however, it contains two complete prophage regions and multiple insertion elements, originating from strains cohabiting with *Lc. paracasei* SRX10 in fermented products (Tables S2 and S3). The spontaneous induction of these prophages in the food matrix could have negative effects on the resident microbiota; thus, future studies will investigate their potential activation at different stages of production. Importantly, no acquired antimicrobial resistance genes were identified in these regions, thus eliminating the possibility of horizontal gene transfer of antibiotic-resistance genes to pathobionts and pathogenic bacteria [65,66]. Previously, resistance against gentamicin, kanamycin, streptomycin, erythromycin, tetracycline, and chloramphenicol was recorded in vitro [7]. This could be attributed to chromosomally encoded antibiotic resistance proteins and, more specifically, to the production of drug efflux pumps, which can remove antibiotics from the bacterial cell. Additionally, this discrepancy could be caused by the intrinsic variability associated with the microdilution method [67], a phenomenon also noted elsewhere [68]. *Lc. paracasei* SRX10 does not code for functional virulence factors. Furthermore, it lacks the machinery for the production of biogenic amines via the decarboxylation of precursor amino acids. The accumulation of biogenic amines in the fermented food matrix can induce adverse effects on the consumer, including intoxication, nausea, abdominal cramps, and headaches [69]. Of note, a protein belonging to the hemolysin III family was annotated in the genome of *Lc. paracasei* SRX10, showing high sequence identity and structural conservation; however, it was previously shown that the strain exhibits γ-hemolytic activity [7]. Although, hemolysin III genes are widespread in lactobacilli, they do not compromise their use in the food chain [70].

The food microbiota plays an important role in the development of the unique sensory profile of fermented products [71]. For community-wide analysis, metataxonomics and metagenomics, as well as conventional microbiological and molecular techniques, are commonly used [72]. However, these methods possess low discriminatory capacity at the strain level [73]. NGS has facilitated the development of accurate methods for strain detection and quantification in fermented products. To this end, we previously developed a multiplex PCR protocol for strain-level detection based on genome-wide sequence polymorphisms [16]. In the present study, we followed the same pipeline to design strain-specific primers for single-step multiplex PCR detection of *Lc. paracasei* SRX10 in monocultures and yoghurt samples. The assay was successful in detecting *Lc. paracasei* SRX10 in yoghurts containing the minimum recommended probiotic or functional concentration [74], thus providing a useful tool for monitoring the presence of the strain of interest in novel fermented products.

## 5. Conclusions

In this study, we examined the genetic basis of the functional, technological, and safety characteristics of *Lc. paracasei* SRX10, a strain originally isolated from traditional Greek cheese and showing biotechnological potential. Genes and genetic clusters involved in survival in the fermented food industry environment were annotated in the genome of the strain. In addition, the metabolic capacity of the strain was predicted using bioinformatic tools to determine its ability to utilize different sugars in the food matrix and to produce compounds that can contribute to the technological and functional character of fermented foods. Regarding the in silico safety assessment, no transferable antibiotic resistance genes or virulence genes were detected in the whole genome of the strain, suggesting that the strain is safe for consumption. Finally, whole-genome-based primers were designed for the efficient and rapid detection of *Lc. paracasei* SRX10 in the food matrix using a single-step multiplex PCR assay.

## Figures and Tables

**Table 1:** Genome characteristics of *Lc. paracasei* SRX10.

Genome Characteristic	Value
Length	2,813,407 bp
GC content	46.40
Total genes	2764
CDSs	2711
rRNAs	6
tRNAs	44
ncRNAs	3
Pseudogenes	106
Contamination (%)	1.89

**Table 2:** Maximum ANI of *Lc. paracasei* SRX10 with strains isolated from dairy products.

*Lc. paracasei* Strain	ANI (%) with SRX10
SRX10	100
KMB_622	99.67
EG9	99.62
FAM18172	99.58
FAM18123	99.51
FAM22279	99.50
SP5	99.42
FAM18110	99.40
Lpp14	99.34
Lpc-yoghurt_C5	99.34
Lpc-yoghurt_C1	99.31
Lpc-yoghurt_C3	99.31
FAM19404	99.26
FAM18175	99.26
ATCC334	99.23
FAM8407	99.20

**Table 3:** Annotation of genes coded by *Lc. paracasei* SRX10 that are implicated in stress tolerance mechanisms.

Locus Tag	Gene Function	Gene	E-Value
	Acid tolerance response		
SRX10_000077	Sodium proton antiporter	*yvgP*	0.0
SRX10_000089	ATP synthase subunit alpha	*atpA*	0.0
SRX10_001052	ATP synthase subunit beta	*atpB*	1.23 × 10^−162^
SRX10_001843	ATP synthase epsilon chain	*atpC*	1.88 × 10^−91^
SRX10_001842	ATP synthase subunit delta	*atpD*	0.0
SRX10_001053	ATP synthase subunit c	*atpE*	2.57 × 10^−37^
SRX10_001054	ATP synthase subunit b	*atpF*	3.59 × 10^−80^
SRX10_001057	ATP synthase gamma chain	*atpG*	1.92 × 10^−211^
SRX10_001055	ATP synthase subunit delta	*atpH*	3.93 × 10^−116^
	Extreme temperature tolerance		
SRX10_002468	Cold shock protein	*cspB*	4.62 × 10^−48^
SRX10_000179	Cold-shock protein	*cspA*	3.08 × 10^−43^
SRX10_001462	Cold shock protein	*cspC*	6.22 × 10^−43^
SRX10_001823	Heat-inducible transcription repressor	*hrcA*	3.76 × 10^−245^
SRX10_000446	Small heat shock protein	*hsp3*	1.48 × 10^−98^
SRX10_ 001002	Small heat shock protein	*hsp1*	7.42 × 10^−112^
SRX10_001824	Gro-P like protein E	*grpE*	3.18 × 10^−127^
SRX10_001825	Heat shock 70 kDa protein	*dnaK*	0.0
SRX10_002247	ATP-dependent protease	*clpC*	0.0
SRX10_000636	Co-chaperonin	*groS*	1.7 × 10^−59^
SRX10_000637	Chaperonin	*groL*	0.0
	Osmotic shock tolerance		
SRX10_002092	Periplasmic glycine betaine choline-binding (lipo)protein	*opuCC*	1.02 × 10^−221^
SRX10_002458	Periplasmic glycine betaine choline-binding (lipo)protein	*choS*	0.0
SRX10_000873	Glycine betaine/carnitine transport ATP-binding protein	*gbuA*	3.07 × 10^−283^
SRX10_000872	Glycine betaine/carnitine transport ATP-binding protein	*gbuB*	5.82 × 10^−189^
	Oxidative stress response		
SRX10_000743	Glutathione peroxidase	*gpo*	9.78 × 10^−112^
SRX10_000517	Thiol-specific peroxidase	*tpx*	8.23 × 10^−117^
SRX10_000197	Peroxidase	*ywbn*	9.75 × 10^−228^
SRX10_000359	Redox regulated molecular chaperone.	*hslO*	2 × 10^−206^
SRX10_002197	NADH dehydrogenase	*ndh*	0.0
SRX10_001933	NADH oxidase	*nox*	0.0
SRX10_001342	NADH oxidase	*nox*	0.0
	Alcohol resistance		
SRX10_002216	Succinate-semialdehyde dehydrogenase	*gabD*	0.0
SRX10_001400	Lactaldehyde dehydrogenase	*aldA*	0.0
SRX10_002037	Glyceraldehyde-3-phosphate dehydrogenase	*gap*	2.1 × 10^−247^

**Table 4:** Annotation of genes encoded by *Lc. paracasei* SRX10 that are implicated in technological characteristics of fermented dairy products.

Locus Tag	Gene Function	Gene	E-Value
Lactose degradation			
SRX10_000981	Galactokinase	*galK*	1.1 × 10^−280^
SRX10_000982	UDP-glucose 4-epimerase	*galE*	4.24 × 10^−247^
SRX10_000983	Galactose-1-phosphate uridylyltransferase	*galT*	0.0
SRX10_000985	Maltose epimerase	*galM*	6.81 × 10^−251^
Fatty acid biosynthesis			
SRX10_001747	Beta-ketoacyl-[acyl-carrier-protein] synthase III	*fabH*	3.96 × 10^−226^
SRX10_001739	Acetyl-CoA carboxylase biotin carboxylase	*accC*	0.0
SRX10_001744	Malonyl CoA-acyl carrier protein transacylase	*fabD*	9.85 × 10^−209^
SRX10_001737	Acetyl-coenzyme A carboxylase carboxyl transferase subunit alpha	*accA*	1.33 × 10^−181^
SRX10_001738	Acetyl-coenzyme A carboxylase carboxyl transferase subunit beta	*accD*	8.74 × 10^−194^
SRX10_001741	Biotin carboxyl carrier protein of acetyl-CoA carboxylase	*accB*	2.52 × 10^−92^
SRX10_000718	Enoyl-(Acyl carrier protein) reductase	*fabG*	3.01 × 10^−166^
SRX10_001063	Enoyl-(Acyl carrier protein) reductase	*fabG*	4.44 × 10^−161^
SRX10_001743	3-oxoacyl-(acyl-carrier-protein) reductase	*fabG*	2.37 × 10^−163^
SRX10_001745	Nitronate monooxygenase	*fabK*	4.56 × 10^−220^
SRX10_001740	3-hydroxyacyl-(acyl-carrier-protein) dehydratase	*fabZ*	3.23 × 10^−98^
SRX10_001742	3-oxoacyl-(acyl-carrier-protein) synthase 2	*fabF*	5.73 × 10^−283^
Proteolysis			
SRX10_002167	Aminopeptidase E	*pepE*	0.0
SRX10_000249	Proline iminopeptidase	*pepI*	4.11 × 10^−223^
SRX10_001793	Xaa-Pro dipeptidase	*pepQ*	2.2 × 10^−274^
SRX10_001533	Proline iminopeptidase	*pepR*	1.97 × 10^−230^
SRX10_002393	X-prolyl dipeptidyl aminopeptidase	*pepX*	0.0
SRX10_001496	Aminopeptidase N	*pepN*	0.0
SRX10_002166	Aminopeptidase C	*pepC*	0.0
SRX10_002386	Proline iminopeptidase	*pepP*	2.92 × 10^−257^
SRX10_000060	Oligopeptidase F	*pepF2*	0.0
SRX10_001991	Oligopeptidase F	*pepF*	0.0
SRX10_001495	PII-type proteinase	*prtP*	0.0
SRX10_001622	Neutral endopeptidase	*pepO*	0.0
SRX10_001991	Oligoendopeptidase F	*pepF*	0.0
SRX10_001034	Dipeptidase	*pepD2*	0.0
SRX10_001432	Dipeptidase	*pepD3*	0.0
SRX10_001528	Dipeptidase A	*pepDA*	0.0
SRX10_002225	Dipeptidase (Serine protease)	*pepD*	0.0
SRX10_001788	Beta-Ala-Xaa dipeptidase	*pepV*	0.0
SRX10_001725	Peptidase T	*pepT*	2.45 × 10^−309^
	Amino acid metabolism		
SRX10_001504	Shikimate dehydrogenase (NADP (+))	*aroE*	8.84 × 10^−211^
SRX10_001502	Shikimate dehydrogenase (NADP (+))	*aroE*	2.98 × 10^−215^
SRX10_001556	Branched-chain amino acid aminotransferase	*ilvE*	8.73 × 10^−262^
EPS biosynthesis			
SRX10_002122	Glycosyl transferase 4-like	*epsD*	1.67 × 10^−96^
SRX10_002127	Capsular polysaccharide biosynthesis protein	*epsB*	4.88 × 10^−141^
SRX10_000238	Exopolyphosphatase	*ppx*	5.77 × 10^−204^
SRX10_000236	Exopolyphosphatase 3	*ppx3*	0.0
SRX10_002126	Tyrosine-protein kinase	*ywqD*	1.54 × 10^−35^
SRX10_002445	Glycosyltransferase like family 2	*epsG*	4.18 × 10^−151^

**Table 5:** Genomic features related to the safety profile of *Lc. paracasei* SRX10.

Feature	
No. of CRISPR arrays	0
Phages	
*Intact*	2
*Incomplete*	0
*Questionable*	1
Mobile elements	0
IS elements	98
Antibiotic resistance genes	
*Perfect hits*	0
*Strict hits*	1
*Loose hits*	210
Virulence genes	0
Biogenic amine genes	0
Plasmids	0

**Table 6:** Primer sets used for the detection of *Lc. paracasei* SRX10.

Primer Code	Sequence (5′-3′)	Product Size (bp)	Reference
3.1F	GTCCGTATAACGAGCCAATGC	137	This study
3.1R	TCGGACGCATACTAGGACAC		This study
1.1F	CGTTAAGTGAAGGCGTAGTCG	534	This study
1.1R	CCACGCACATGCTATTCTAGTG		This study
LacF	AGCAGTAGGGAATGTTCCA	340	[43]
LacR	ATTYCACCGCTACACATG		[43]

## Data Availability

The WGS of *Lc. paracasei* SRX10 was deposited at DDBJ/ENA/GenBank under the accession JAXBDF000000000. The version described in this paper is version JAXBDF010000000.

## Supplementary Materials

The following supporting information can be downloaded at: https://www.mdpi.com/article/10.3390/microorganisms12010093/s1, Figure S1: (A) Pangenome analysis of *Lc. paracasei* strains isolated from fermented milk products. The absence (white) and presence (dark blue) of core and accessory genes are depicted in the matrix. Highlighted in red are clusters assigned to *Lc. paracasei* SRX10. (B) Pie chart depicting the categorization of genes into the core, soft-core, shell, and cloud genomes and the number of isolates in which they are present; Figure S2: Examination of the features of the hemolysin III family protein annotated in the genome of *Lc. paracasei* SRX10 using InterPro; Table S1: Classification of annotated proteins into COGs; Table S2: CAZyme annotation of proteins encoded by *Lc. paracasei* SRX10; Table S3: Annotation of prophage regions in the genome of *Lc. paracasei* SRX10; Table S4: Insertion elements contained in the genome of *Lc. paracasei* SRX10.
