# Supplementary material for: Dissecting the Genetic Basis of the Technological, Functional, and Safety Characteristics of Lacticaseibacillus paracasei SRX10
Source: Microorganisms. 2024 Jan 2;12(1):93. doi: 10.3390/microorganisms12010093 (PMC10820299; doi:10.3390/microorganisms12010093)
Supplement: Supplementary file 1 [file microorganisms-12-00093-s001.zip › Figure S2.pdf]

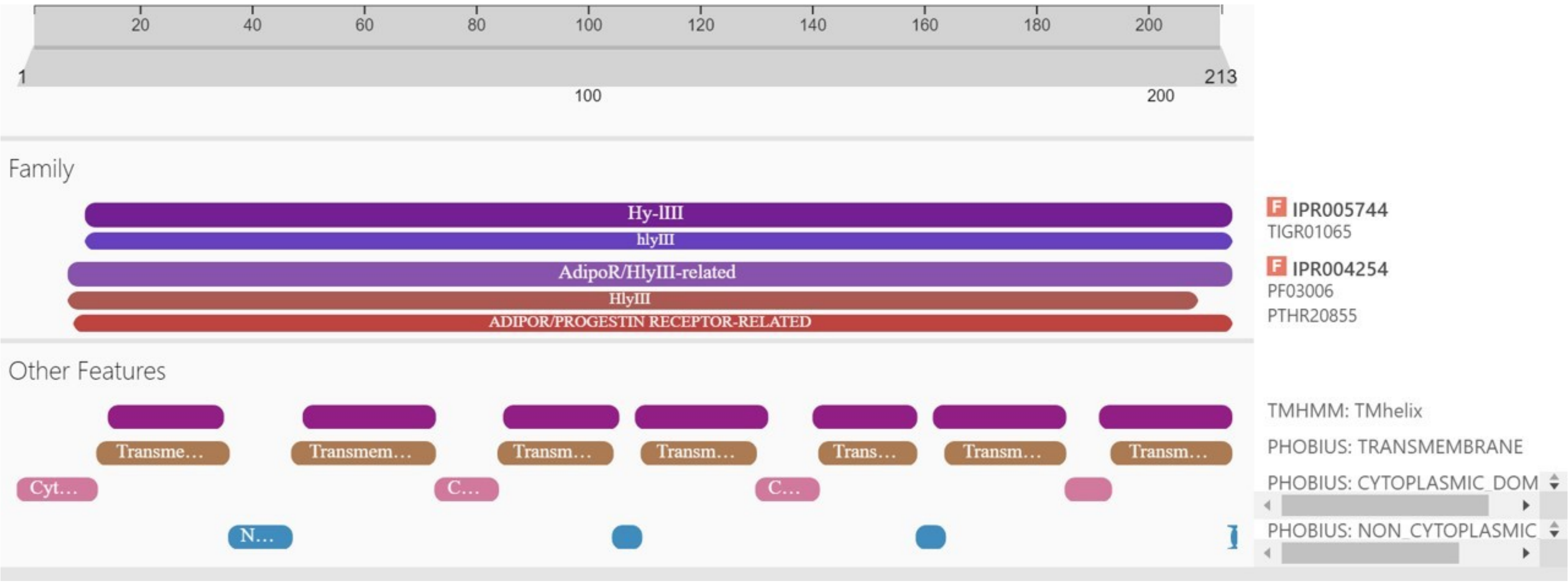

Family
Hy-lIII
hlyIII
AdipoR/HlyIII-related
HlyIII
ADIPOR/PROGESTIN RECEPTOR-RELATED
IPR005744
TIGR01065
IPR004254
PF03006
PTHR20855
Other Features
TMHMM: TMhelix
PHOBIUS: TRANSMEMBRANE
PHOBIUS: CYTOPLASMIC_DOM
PHOBIUS: NON_CYTOPLASMIC
