# Supplementary figures and images for: Dissecting the Genetic Basis of the Technological, Functional, and Safety Characteristics of Lacticaseibacillus paracasei SRX10
Source: Microorganisms. 2024 Jan 2;12(1):93. doi: 10.3390/microorganisms12010093 (PMC10820299; doi:10.3390/microorganisms12010093)

A

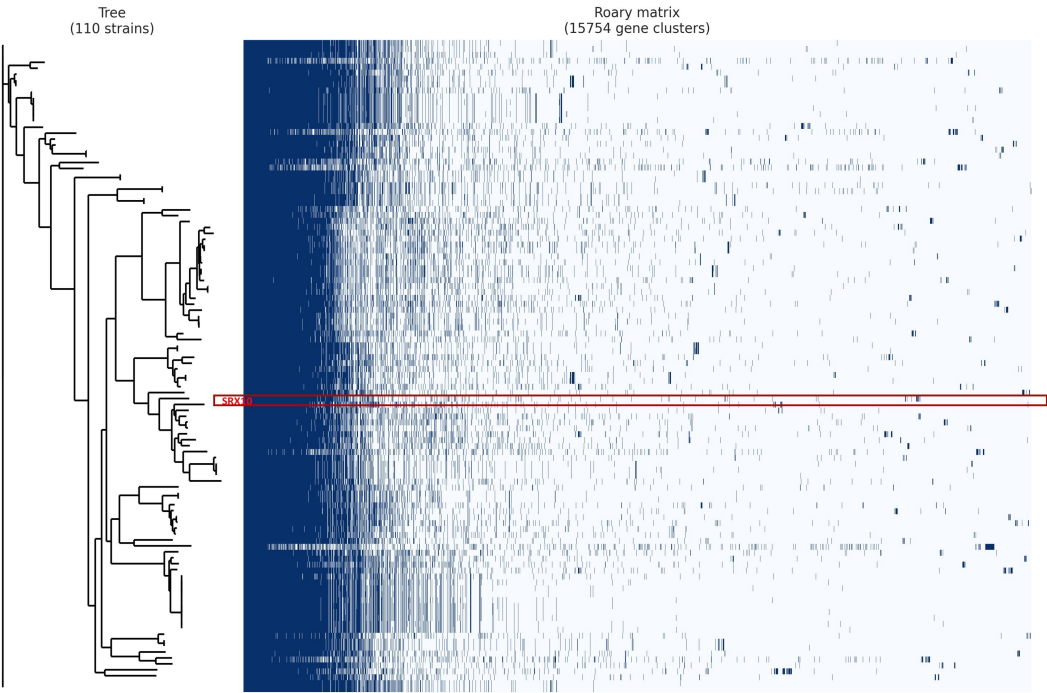

B

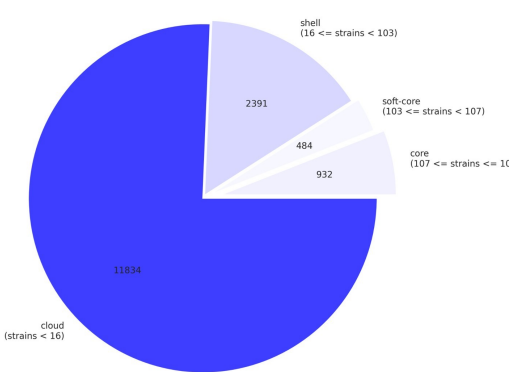

Supplement: Supplementary file 1 [file microorganisms-12-00093-s001.zip › Figure S1.pdf]
